# Preparation and Properties of Thermoplastic Polyurethane Composites Filled with Powdered Buckwheat Husks

**DOI:** 10.3390/ma15010356

**Published:** 2022-01-04

**Authors:** Marcin Włoch, Paulina Landowska

**Affiliations:** Department of Polymers Technology, Faculty of Chemistry, Gdańsk University of Technology, Gabriel Narutowicz Str. 11/12, 80-233 Gdańsk, Poland; paula.landowska@gmail.com

**Keywords:** thermoplastic polyurethane, buckwheat husks, composites, morphology, mechanical properties, dynamic-mechanical properties

## Abstract

Bio-based fillers for the polymer composites are still interesting from the scientific and industrial point of view, due to their low cost and renewable nature. In this work partially green composites were obtained by the mixing of thermoplastic poly(ester-urethane) with the unmodified and modified (by acetylation) grinded buckwheat husks. Obtained biocomposites were characterized in the terms of their chemical structure (FTIR), microstructure (SEM), thermal stability (TGA), thermomechanical properties (DMTA), and selected mechanical properties. The results showed that introduction of grinded buckwheat husks (even if the amount is 60 wt%) permit retaining high values of tensile strength (around 8–10 MPa), but the increasing amount of applied filler is connected with the decreasing of elongation at break. It can result from good interaction between the polymer matrix and the bio-based filler (confirmed by high values of polymer matrix-filler interaction parameter determined from Pukánszky’s model for the tensile strength of composites). The applied chemical treatment results in changing of mechanical properties of filler and composites. Obtained results confirmed the possibility of using powdered buckwheat husks as filler for thermoplastic polyurethane.

## 1. Introduction

By-products from the agricultural industry are generally the source of bio-based fillers, which can be applied to the production of polymer-based composites. The chemical modification of fillers can improve the mechanical performance of polymer composites. Several different chemical modifications were proposed for the bio-based fillers, e.g., mercerization (using sodium hydroxide), acetylation (using acetic anhydride and acetic acid), oxidation (using potassium permanganate), grafting (by ring opening polymerization, living polymerization or using coupling agents), and others. The procedure of single chemical modification can be different for different types of bio-based fillers and it results from the different chemical composition, size, surface characteristic, and mechanical properties of filler subjected to the process [1,2,3].

The green thermoplastic polyurethane materials can be obtained using bio-based substrates for the synthesis of thermoplastic polyurethanes (including diisocyanates, polyols or/and low-molecular weight chain extenders) or/and using bio-based fillers. Bio-based TPU can be synthesized using, for example: fatty acids derivatives [4], lysine [5], isosorbide [6,7]. Several different bio-based fillers were proposed for the polyurethanes, for example: hemp fibers [8], flax fibers [9], kenaf fibers [10], sugar palm fibers [11], roselle fibers [12], tea waste fibers [13], and cocoa pod husk fibers [14]. 

Rice and buckwheat husks are examples of agriculture by-products that can be applied as a fillers in the preparation of polymer composites. In the area of polyurethane-based biocomposites special place is occupied by rice husks. Rozman et al. [15] described poly(ether-urethane)s filled with rice husks. Composites were prepared by the reaction of bio-based filler with 4,4′-diphenylmethane diisocyante followed by the reaction with poly(ethylene glycol). The effect of rice husks amount and size (150–180, 180–250 or 250–500 μm) on the flexural, tensile, and impact properties were investigated. Sherif et al. [16] studied the effect of surface treatment (using steam or sodium hydroxide solution) of ground rice husk (with average particle size less than 200 μm) on the polyurethane prepared using castor oil and polymeric diphenylmethane diisocyanate (PMDI). The steam treatment improved the interfacial adhesion within the composite, which was connected with enhanced hardness of the material. da Silva et al. [17,18] investigated polyurethane foams based on modified tung oil and reinforced with rice husk ash. Rice husks are also used in other polymers (for example polyethylene, polypropylene, poly(lactic acid)) like a filler [19].

Buckwheat husks, which are considered in this work, are another example of bio-based filler that can be used for the preparation of polymer composites. Andrzejewski et al. [20] obtained polyethylene and poly(lactic acid)-based biocomposites filled with buckwheat husk filler (materials contained from 1 to 25 or 30 wt% of the filler for PLA and PE, respectively) by rotational molding. Andrzejewski, Barczewski, and Szostak [21] obtained polypropylene-based composites with buckwheat husk filler (materials contained 10, 30, and 50 wt% of the filler). Authors also proved that effectiveness of the composite constituents compatibility can be improved by using maleic anhydride grafted polypropylene.

The main aim of this work was to obtain thermoplastic polyurethane-based composites filled with powdered buckwheat husks. The bio-based filler was used unmodified and chemically modified by the acetylation. Thermoplastic polyurethane was synthesized by prepolymer method using 4,4′-diphenylmethane diisocyanate, ester-based oligomeric diol, and 1,4-butanediol. Composites, prepared using a Brabender mixer, contained 10, 20, 30, 40, 50, or 60 wt% of bio-based filler. Obtained materials were characterized in the terms of their chemical structure (FTIR), microstructure (SEM), viscoelastic properties (DMTA), thermal stability (TGA), and mechanical properties (static tensile test, Shore hardness test).

## 2. Materials and Methods

### 2.1. Synthesis of Thermoplastic Polyesterurethane

**Figure 1:**
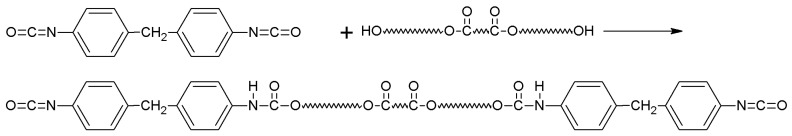
Synthesis of prepolymer from aromatic diisocyanate and ester-based polyol.

Thermoplastic polyurethane was prepared using two-step method, also called prepolymer method. In the first step (Figure 1), urethane prepolymer was synthesized using 4,4′-diphenylmethane diisocyanate (MDI, BorsodChem, Kazincbarcika, Hungary) and ester-based polyol, i.e., α,ω-oligo(ethylene-butylene adipate)diol (POLIOS 55/20, Purinova, Bydgoszcz, Poland), which is characterized by average molecular weight around 2000 g mol^−1^, hydroxyl number 58 mg KOH g^−1^ and acid number 0.3 mg KOH g^−1^. The synthesis of prepolymer was carried out at 80 °C for 2 h (under the vacuum) and resulted polymer was characterized by the amount of unreacted isocyanate groups equal 7.2 wt%. 

**Figure 2:**
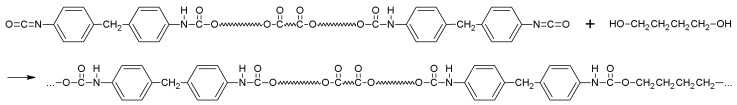
Reaction of the prepolymer with low-molecular weight chain extender, i.e., 1,4-butanediol.

In the next step (Figure 2) prepolymer was reacted with chain extender, i.e., 1,4-butanediol (Brenntag, Kędzierzyn-Koźle, Poland). The reaction was catalyzed using 1,4-diazabicyclo[2.2.2] octane (DABCO, Sigma Aldrich, Saint Louis, MO, USA), which was previously dissolved in the chain extender (to obtain 0.3 wt% solution of DABCO in BDO). The molar ratio of [NCO]/[OH] groups during prepolymer chains extending step was equal 0.95). Finally, obtained material was seasoned at 100 °C for 24 h.

### 2.2. Chemical Modification of Powdered Buckwheat Husks

**Figure 3:**
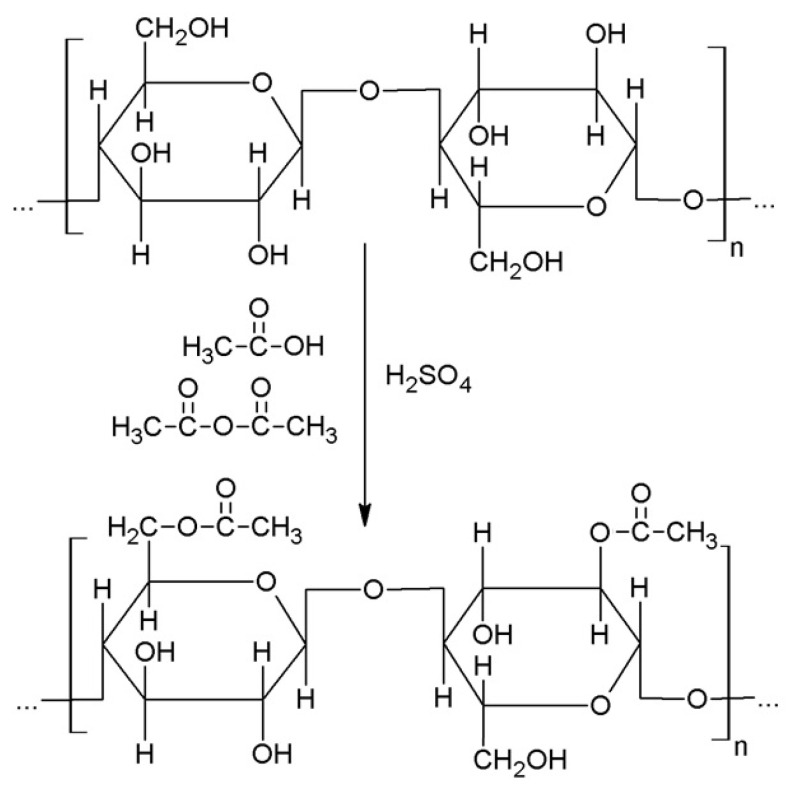
Chemical modification of cellulose-based filler.

The acetylation of grinded buckwheat husks was performed by the soaking of 50 g of filler in solution containing 200 mL of acetic anhydride, 198 mL of acetic acid, and 2 mL of concentrated sulphuric(VI) acid. The soaking time was equal 2 days and after that the filler were washed with distilled water several times until natural pH was obtained. The filler were dried for 48 h at 80 °C and 24 h at 100 °C. The unmodified filler was dried for 24 h at 100 °C. The general scheme of acetylation of cellulose-based filler is presented in Figure 3. The performed chemical treatment involves the reaction of primary and secondary hydroxyl groups with acetic anhydride.

### 2.3. Preparation of Thermoplastic Polyurethane Composites with Powdered Buckwheat Husks

The small pieces of TPU and bio-based filler, i.e., unmodified (BWH) and modified (BWH-Ac) grinded buckwheat husks, were mixed using a Brabender mixer at 170 °C, for 10 min with mixing speed equal 100 rpm. After that, the obtained materials were pressed by using a hydraulic press in two steps: (I) 170 °C, 5 MPa, 5 min and (II) 21 °C, 5 MPa, 5 min. The reference sample (TPU-REF) without fillers was also prepared by the same procedure. The sample code indicates the type (unmodified, BWH or modified buckwheat husks powder, Ac-BWH) and amount (in wt%) of applied filler, for example: sample coded as TPU-BWH-60 is composites filled with unmodified powdered buckwheat husks, which amount in the material is equal 60 wt%.

### 2.4. Testing Methods

Chemical structure of fillers, thermoplastic polyurethane, and composites was verified by Fourier Transform Infrared Spectroscopy (FTIR) using Nicolet 8700 FTIR Spectrophotometer (ThermoElectron Co., Waltham, MA, USA). Spectra were registered at room temperature for wave numbers between 500 and 4000 cm^−1^ at nominal resolution 4 cm^−1^. Each spectrum is an average from 64 scans.

Microstructure of cross-sections of prepared composites was studied using a Phenom G2 PRO scanning electron microscope (Phenom World, Eindhoven, The Netherlands). The micrographs were prepared using desktop scanning electron microscope with accelerating voltage of 5 kV. Unmodified and chemically modified powdered buckwheat husks were analysed using Quanta FEG scanning electron microscope equipped with the Everhart–Thornley detector (ETD) and accelerating voltage of 10 kV.

Dynamic mechanical thermal analysis (DMA) was carried out using a DMA Q800 analyzer (TA Instruments, New Castle, Delaware, USA) in a temperature range from −100 to +100 °C at heating rate of 4 °C min^−1^. The measurements were performed in three-point bending mode at operating frequency 1 Hz under nitrogen atmosphere. The dimensions of the composites samples subjected to the test were 10 mm (width) × 40 mm (length) × 2 mm (thickness). The variation of storage modulus (E′), loss modulus (E″) and damping factor (tan δ) vs. temperature was determined. Glass transition temperature (T_g_) of prepared materials was determined as temperature at which tan δ reaches maximum value.

Thermal stability of materials were determined using TGA Pyris 1 (Perkin Elmer, Waltham, MA, USA) analyzer. Weight of the sample was around 5–10 mg. The samples were heated from 50 to 600 °C at a rate of 20 °C min^−1^ under inert atmosphere (nitrogen).

Selected mechanical properties (tensile properties and hardness) were characterized for the prepared thermoplastic polyurethane and polyurethane composites with powdered buckwheat husks. Tensile properties (i.e., tensile strength and elongation at break) were determined using universal testing machine Zwick/Roell Z020 and dumbbell specimens, according to ISO 527-2 standard. The tensile speed was 200 mm/min and presented values are averages from five independent tests. Hardness was determined using an electronic Shore type A Durometer (Zwick/Roell) according to ISO 868 standard and presented results are averages from random five independent measurements (different places at the surface of the same sample).

## 3. Results and Discussion

### 3.1. Verification of Chemical Structure

**Figure 4:**
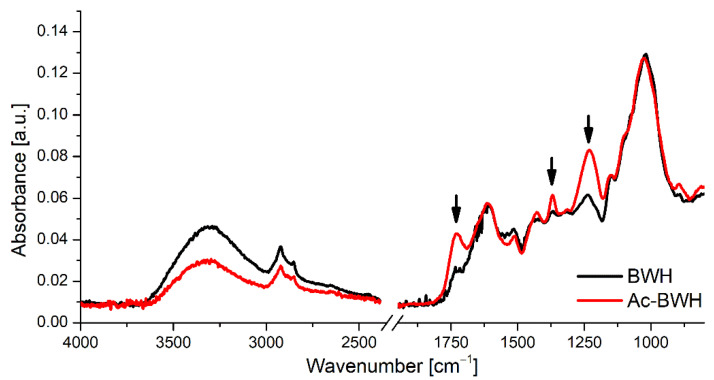
FTIR spectra of unmodified (BWH) and modified (Ac-BWH) powdered buckwheat husks (arrows indicate bands related to acetyl groups).

Figure 4 presents FTIR spectra of unmodified and acetylated buckwheat husks. The maxima of bands corresponding to the acetyl groups are observed at 1726, 1369, and 1230 cm^−1^, and can be assigned to carbonyl stretching vibration, methyl in-plane bending, and C–O stretching, respectively. Ohkoshi [22] observed bands with similar maxima (i.e., 1732, 1369 and 1230 cm^−1^ for acetylated wood. Zhou et al. [23] indicated that for acetylated cellulose the maxima of bands related to acetyl groups are observed at 1751 (vC=O), 1371 (vC–CH_3_), and 1235 cm^−1^ (vC–O).

**Figure 5:**
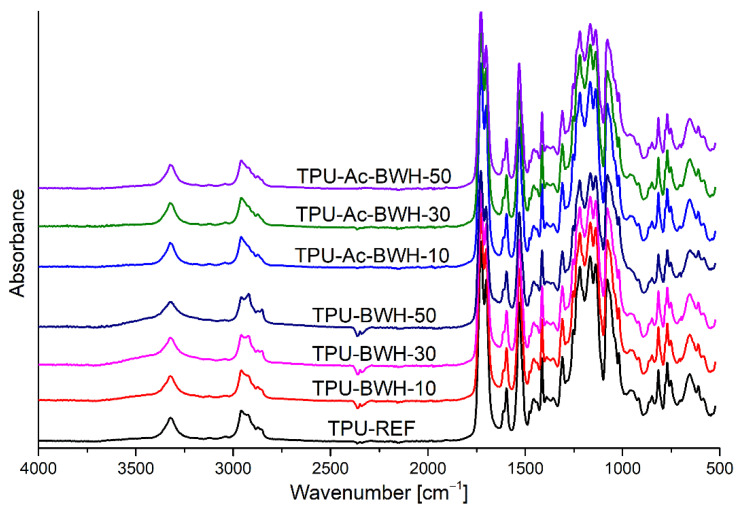
FTIR spectra of thermoplastic polyurethane and composites with bio-based filler.

Figure 5 presents FTIR spectra of thermoplastic polyurethane (TPU-REF) and TPU composites with unmodified (TPU-BWH-10, TPU-BWH-30 and TPU-BWH-50).and acetylated buckwheat husks (TPU-Ac-BWH-10, TPU-Ac-BWH-30 and TPU-Ac-BWH-50). In FTIR spectrum of TPU, the absorption band at 3323 cm^−1^ corresponds to stretching vibrations of hydrogen-bonded amine groups (presented in urethane moieties). The double peak with maxima at 1728 and 1701 cm^−1^ is related to stretching vibrations of free and hydrogen bonded carbonyl groups (presented in soft segments and urethane moieties), respectively. The absorbance of peaks related to carbonyl stretching vibrations is not affected by the introduction of powdered buckwheat husks.

The applied chemical treatment results in decreasing of hydroxyl group content in the chemical structure of bio-based filler (the hydroxyls presented in the cellulose, hemicellulose, and lignin are reacted with acetic anhydride to form acetyl groups). The broad peak visible in FTIR spectra of obtained materials (mainly in composites contained unmodified buckwheat husks) related to stretching vibrations of –OH groups (3200–3600 cm^−1^) confirm that in the chemical structure of modified filler was replaced by acetyl –O-C(O)–CH_3_ groups during chemical treatment. In general, the FTIR spectra of TPU and TPU-based composites with BWH and Ac-BWH are very similar.

### 3.2. Microstructure

**Figure 6:**
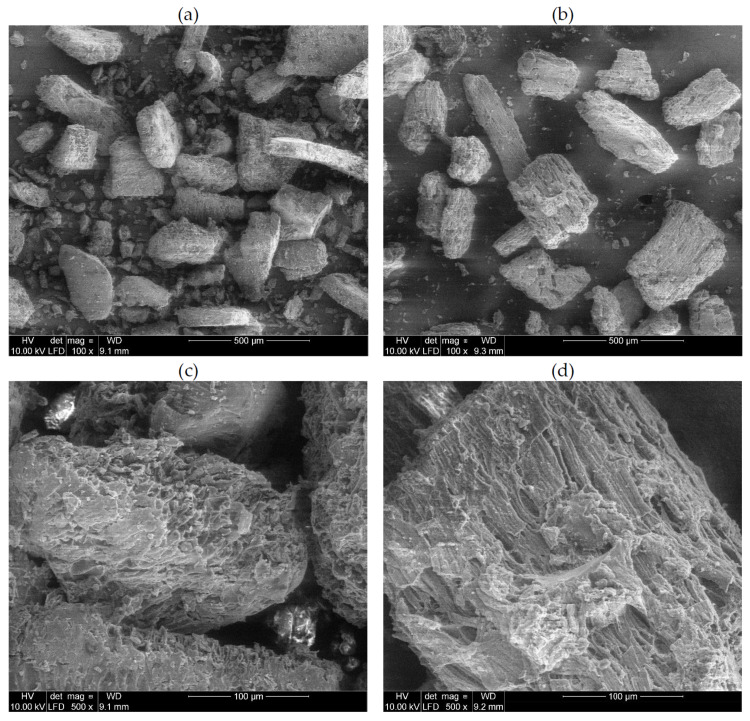
SEM micrographs powdered buckwheat husks: (**a**,**c**) BWH and (**b**,**d**) Ac-BWH.

SEM micrographs of unmodified and acetylated buckwheat husks are presented in Figure 6. The applied bio-based filler has plate shape. In general, the thickness of the plates is around 30–60 μm, while their width is in the range from 50 to 300 μm. The applied chemical treatment results in increasing of particles roughness without significant changes of their sizes. Buson et al. [24] found that acetylation of bamboo fibers results in increasing of their surface roughness, which is connected to removal of waxes and oils that fill the some spaces in the microstructure of fibers. Kabir et al. [25] also observed increasing of surface roughness for fibers subjected to acetylation.

**Figure 7:**
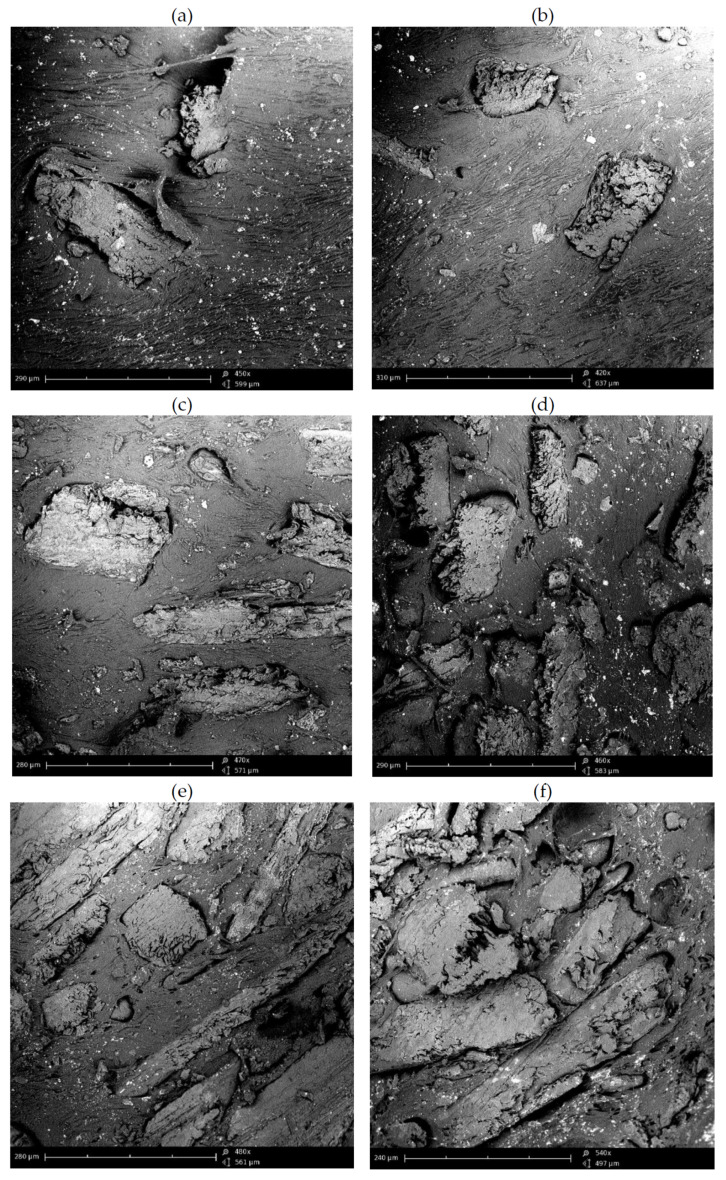
SEM micrographs of cross-sections of thermoplastic polyurethane-based composites with powdered buckwheat husks: (**a**) TPU-BWH-10, (**b**) TPU-Ac- BWH-10, (**c**) TPU-BWH-30, (**d**) TPU-Ac-BWH-30, (**e**) TPU-BWH-50, and (**f**) TPU-Ac-BWH-50.

SEM micrographs of cross-sections of prepared composites with unmodified and acetylated buckwheat husks are presented in Figure 7. Generally good adhesion between polyurethane matrix and bio-based filler is visible, but this behavior is not homogenous probably due to applied cutting technique. The applied chemical treatment changes the mechanical properties and microstructure of surface of bio-based filler. The surface of cross-sections of composites filled with acetylated buckwheat husks powder is more irregular and rough.

### 3.3. Thermomechanical Properties

**Figure 8:**
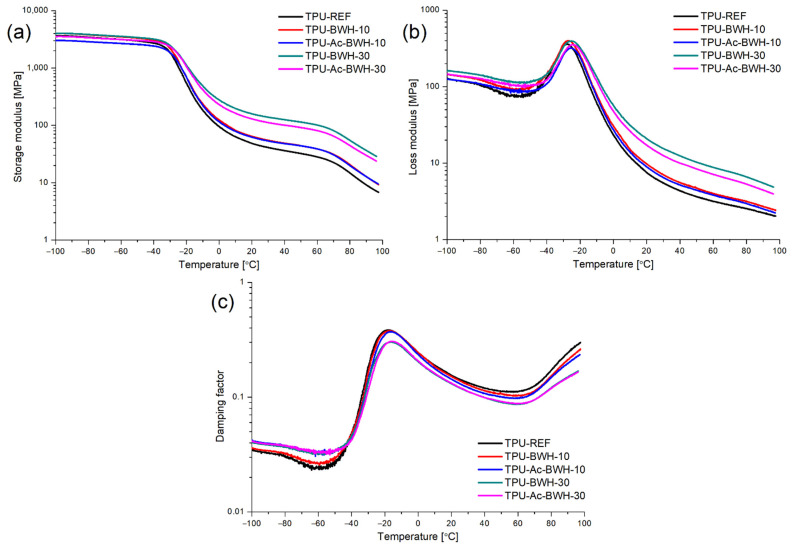
(**a**) Storage modulus, (**b**) loss modulus and (**c**) damping factor vs. temperature for thermoplastic polyurethane and composites filled with unmodified and modified buckwheat husks powder.

The temperature dependence of storage modulus (E′), loss modulus (E″), and damping factor (tan δ) is presented in Figure 8, while values of some parameters characterized viscoelastic properties of composites are presented in Table 1 and Table 2. The highest values of storage modulus are observed in glassy state (below T_g SS_) and during the glass transition of soft segments significant decreasing of this parameter is noted as a result of significant increase in the mobility of polymer chain segments during glass transition. At room temperature, where composites are in rubbery state, storage modulus reach relatively low values (47–156 MPa), but obtained results suggest that introduced bio-based filler improves materials stiffness (as a result of E′ values increasing). Decreasing of E′ at room temperature suggest that applied chemical treatment decreasing the stiffness obtained composites with treated filler in comparison to composites with untreated filler. Another decreasing of storage modulus is observed above 60 °C which can be related to the glass transition temperature of hard segments (T_g HS_). According to the literature T_g HS_ of thermoplastic polyurethanes is observed below 100 °C, while the melting occurs above 100 °C [26]. Increasing of loss modulus during the glass transition is connected to increasing of energy dissipation caused by improved polymer chains mobility. The energy is dissipated in the molecular rearrangements, which lead to a permanent and non- reversible deformation [27]. The maximum value of E″ observed for the composites is increasing in order TPU-Ac-BWH < TPU < TPU-BWH.

The glass transition temperature slightly decreasing when amount of the bio-based filler in the composite is increasing, which suggest that polymer chains have higher mobility. The applied chemical modification also results with decreasing of T_g SS_ values (Table 2). It can be resulted from the increasing of hydrophobicity of filler during chemical treatment, which decreasing interaction between polymer matrix and bio-based filler. The highest values of damping factor (tanδ) were observed for the composites containing 10 wt% of powdered buckwheat husks, while the lowest for composites containing 30 wt% of bio-based filler. It suggests that introduction of higher amounts of BWH or Ac-BWH results in decreasing of damping properties of obtained materials. Generally, materials characterized by tanδ > 0.3 over the temperature range of at least 60–80 °C can be used in real damping applications [28].

### 3.4. Thermal Stability

**Figure 9:**
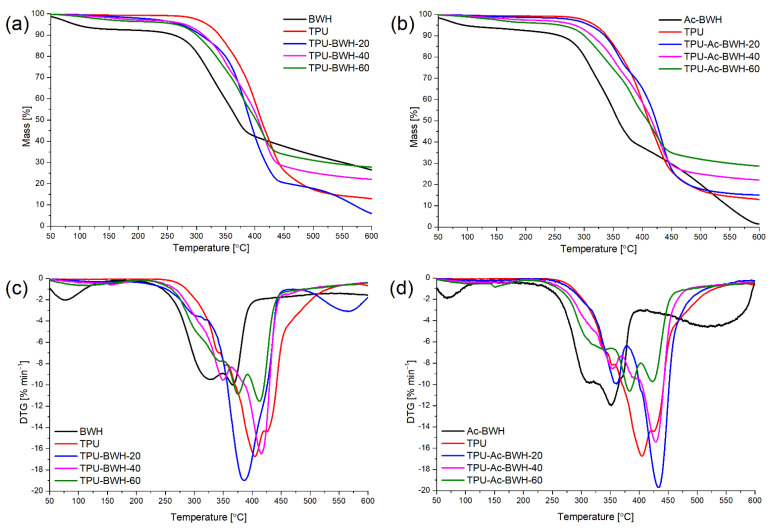
TG and DTG curves of (**a**,**c**) unmodified buckwheat husks, thermoplastic polyurethane and their composites and (**b**,**d**) chemically modified buckwheat husks, thermoplastic polyurethane, and their composites.

Parameters related to thermal stability of unmodified and acetylated powdered buckwheat husks, and composites containing them are presented in Table 3, while thermogravimetric (TG) and derivative thermogravimetric (DTG) curves are presented in Figure 9. It is clearly visible that thermal stability of thermoplastic polyurethane is higher than in the case of used bio-based fillers. The applied chemical modification method has not significantly changed the thermal stability of the filler. Buckwheat husks are composed mainly of carbohydrates (more than 90 wt%), while proteins and fats are presented in small amounts [29]. The carbohydrates presented in high amounts in buckwheat husks are lignin (>30 wt%), cellulose (>30 wt%), and hemicellulose (>10 wt%) [30,31]. The thermal decomposition (in inert atmosphere) of powdered buckwheat husks is a multi-stage process which comprise degradation of all constituents (lignin, cellulose, and hemicellulose), but main stages are related to main carbohydrate components. The thermal decomposition of pure hemicellulose occurs in the temperature range from 220 to 315 °C with the maximum rate at 260 °C, but with the mixture with cellulose the temperature of maximum degradation rate is shifted to higher temperatures. The thermal degradation of cellulose occurs in 315–390 °C with the maximum rate at 355 °C. Lignin thermal decomposition is observed from 220 to 500 °C and the maximum rate of degradation is observed at the same temperature like for cellulose [32].

In the applied bio-based filler thermal degradation mostly occurs in the range 200–400 °C, and in this range two maxima are observed: one related to the thermal decomposition of hemicellulose (310–330 °C) and second one to thermal decomposition of cellulose and lignin (350–370 °C). The applied chemical modification results in the decreasing of temperatures at which occur degradation of mentioned constituents probably due to acidic nature of substrates used for the acetylation of buckwheat husks, which may cause degradation (etching) of microstructure of filler and its constituents (dissolving and further removing). The mentioned effects can be also confirmed by third step (400–600 °C) of degradation of observed only for acetylated buckwheat husks powder.

Thermal decomposition of synthesized thermoplastic polyurethane can be described as one-step process, which is typical for the polyurethanes without or with low degree of phase separation (commonly observed for cross-linked and some thermoplastic ones). The temperature at which degradation rate reaches maximum value was around 405 °C, and that value is in the rage 380–420 °C, which was observed by Datta et al. [33] for polyurethanes synthesized using MDI, POLIOS 55/20 and BDO by prepolymer method (percentage content of the isocyanate groups was equal 6% in prepolymer). Typical hard segments of TPUs are made from diisocyanates and low molecular chain extenders, while soft segments are consisted of polyols. The formation of microphase-separated structures is a result of hard and soft segments incompatibility (due to different chemical structure and mobility in the system). The factors that affects formation of micro-phase separated systems are chemical structure of reagents (including symmetry and polarity), polymerization method (one- or two-step method), further processing of material (melting, mixing and cooling condition) and others [34]. If synthesized polyurethanes have microphase-separated structure, the thermal decomposition occurs in the two steps. The first stage is connected with thermal dissociation of the hard segments (urethane moieties) in the polyurethane, while the second peak is connected with the thermal decomposition of soft segments contained commonly ester (like in the case of use POLIOS 55/20 as polyol [33,35] or other ester-based polyol) or ether bonds.

Generally increasing amount of filler in polymer matrix results in decreasing of thermal stability of TPU-based composites, as a result of presence of bio-based filler characterized by lower thermal stability. It can be stated that thermal decomposition of prepared composites occurs in two stages, where first one is related to the degradation of powdered buckwheat husks, while the second one is connected to the thermal decomposition of polymer matrix. In the case of composites containing at least 40 wt% of filler another step is also visible at the beginning of thermal decomposition, probably due to the fact the filler becomes predominant constituent in the microstructure of the composite.

### 3.5. Selected Mechanical Properties

The selected mechanical properties of synthesized TPU and TPU composites are presented in Table 4. The increasing amount of powdered buckwheat husks in the composites results in decreasing of elongation at break, which is commonly observed trend for the composites with particulate bio-based fillers [13,20,36,37]. Applied modification of the filler results in higher values of elongation at break, when the filler is presented in the amount 10–20 wt% in composites. It can be resulted from the surface degradation (followed by it softening) of the filler during the chemical modification. Tensile strength of obtained composites is comparable to the values observed for thermoplastic polyurethane, but higher values are observed for the composites with unmodified powdered buckwheat husks (contained 40–60 wt% of the filler). Buson et al. [24] investigated mechanical properties of acetylated bamboo fibers and found that applied chemical treatment results in increasing of tensile modulus and decreasing of tensile strength and elongation at break, in comparison to unmodified fibers. This behavior is resulted from the decreasing of the intermolecular interactions of the filler constituent macromolecules (caused by the reaction of hydroxyl groups of cellulose, lignin and hemicellulose with acetic anhydride). The lower mechanical properties of filler results in lower mechanical properties obtained composites, but at the highest loading of acetylated buckwheat husks powder (i.e., 60 wt%) mechanical properties of composite are similar to that observed for composites with unmodified filler.

Hardness of composites increasing with increasing amount of introduced filler, which is resulted from higher hardness and stiffness of applied filler in comparison to polymer matrix.

The effectiveness of stress transfer between the polyurethane matrix and buckwheat husk filler can be improved by the interaction of filler with polymer matrix. The relationship between the tensile strength of polymer composites and interfacial interactions was described by Pukanszky [38] and can be calculated as follows:

(1) $$\sigma_{c}=\sigma_{m}\cdot \left(\frac{1-V_{f}}{1+2.5\cdot V_{f}}\right)\cdot e^{B\cdot V_{f}}\;$$

where: σ_c_ is the tensile strength of the prepared composites, σ_m_ is the tensile strength of the thermoplastic polyurethane, B is the parameter related to the matrix–filler interactions (which is related to size of interface and properties of the interphase), V_f_ is the filler volume fraction. V_f_ is calculated using following equation:

(2) $$V_{f}=\frac{\rho_{c}}{\rho_{f}}\cdot w_{f}\;$$

where: ρ_c_ is the density of the prepared composites, ρ_f_ is the density of the filler, w_f_ is weight fraction of the filler in the composite.

Equation (1) should be rewritten in the following way:

(3) $$\frac{\sigma_{c}\left(1+2.5\cdot V_{f}\right)}{\sigma_{m}\left(1-V_{f}\right)}=e^{B\cdot V_{f}}$$

(4) $$\ln\frac{\sigma_{c}\left(1+2.5\cdot V_{f}\right)}{\sigma_{m}\left(1-V_{f}\right)}=B\cdot V_{f}$$

and $\ln\frac{\sigma_{c}\left(1+2.5\cdot V_{f}\right)}{\sigma_{m}\left(1-V_{f}\right)}\;$ should be plotted against V_f_. The slope of a straight line is interaction parameter B.

The densities of the samples, filler volume fractions and matrix–filler interaction parameters (calculated from Pukanszky relationship) for the prepared TPU-based composites filled with unmodified and modified are presented in Table 5. The values of polymer matrix-filler interaction parameters suggest that chemical treatment applied for filler results in worsening of matrix-filler interaction (probably due to decreasing hydrophilicity of the filler after the treatment). On the other hand, observed values of B parameter are relatively high in comparison to this reported in the literature for other polymer composites, for example: poly(lactic acid)/milled sunflower husks (B = 2.50; 2.59), glycol modified poly(ethylene terephthalate)/milled sunflower husks (B = 2.04; 2.57), polystyrene/milled sunflower husks (B = 2.56; 2.65) [39], polypropylene/unmodified wood flour (B = 1.8), polypropylene/wood flour modified with maleic anhydride (B = 1.1) [40]. The matrix–filler interaction parameter is affected by several different factors, i.e., the size of the interface (connected to the specific surface area of the filler), surface treatment method, aggregation, and anisotropy of the filler and polymer matrix chemical structure and properties [38]. In the case of obtained composite one of the most important factor is polarity of the thermoplastic polyurethane and applied bio-based filler (consisted mostly of carbohydrates).

## 4. Conclusions

The obtained results confirmed that powdered buckwheat husks can be used as a filler for thermoplastic polyurethanes. High values of tensile strength (around 8–11 MPa) can be achieved even when the amount of bio-based filler is equal 50–60 wt%, which results from good interaction between polymer matrix and filler confirmed by high value of B parameter (B = 3.89) determined using Pukánszky model related to tensile strength of polymer composites. Unfortunately increasing content of the filler in the composite results in decreasing of elongation at break, which commonly observed trend in polymer biocomposites. The applied chemical treatment (acetylation using acetic anhydride, acetic acid, and acidic catalyst) decreasing hydrophilicity of the filler, which weakening the interaction between polymer matrix and carbohydrate-based filler (B = 3.67) which is connected with decreasing of tensile strength and increasing of elongation at break. The stiffness (connected with values of storage modulus at room temperature) of the materials increasing with increasing amount of filler and higher values were observed for unmodified buckwheat husk powder. Thermal stability of obtained materials is negligible lower in comparison to unfilled TPU, which is resulted from lower thermal stability of bio-based filler (which degradation begins at around 200 °C).

TPUs filled with bio-based fillers (including particulate fillers and fibres) can be used for several applications in construction, automotive, and sports industries. Prepared partially green composites extend the group of materials that can be used in listed above areas, but additional tests are required to confirm suitable performance for specific applications.

## Figures and Tables

**Table 1:** Selected thermomechanical properties of thermoplastic polyurethane and composites containing unmodified and modified buckwheat husks powder.

Material Code	Storage Modulus at −100 °C, MPa	Storage Modulus at +21 °C, MPa	Maximum Value of Loss Modulus, MPa	Temperature at Which E″ Reached Maximum Value, °C
TPU-REF	3647	47	360	−28.0
TPU-BWH-10	4025	63	399	−26.7
TPU-BWH-30	3012	156	394	−25.1
TPU-Ac-BWH-10	4024	61	319	−25.5
TPU-Ac-BWH-30	3541	127	353	−24.2

**Table 2:** Glass transition temperature and related to this temperature damping factor value for thermoplastic polyurethane and composites containing buckwheat husks powder.

Material Code	Glass Transition Temperature of Soft Segments T_g SS_, °C	Tan Delta at T_g_
TPU-REF	−17.9	0.3480
TPU-BWH-10	−16.9	0.3784
TPU-BWH-30	−16.2	0.3010
TPU-Ac-BWH-10	−16.6	0.3710
TPU-Ac-BWH-30	−15.5	0.3055

**Table 3:** Parameters related to thermal stability of powdered buckwheat husks and obtained thermoplastic poly(ester-uretane)s filled with bio-based filler.

Material Code	T_d 5%_, °C	T_d 10%_, °C	T_d 50%_, °C	T_d max_, °C	Char Yield at 600 °C, %
BWH	91.8	260.0	370.4	327.8/366.4	26.5
BWH-Ac	93.3	259.1	358.0	313.6/352.0	1.4
TPU-REF	318.5	338.7	410.8	404.7	13.0
TPU-BWH-20	272.5	307.3	391.4	386.4	6.0
TPU-BWH-40	280.2	311.8	405.5	349.2	22.1
TPU-BWH-60	264.3	300.1	401.8	375.8	27.8
TPU-Ac-BWH-20	309.1	335.7	423.2	361.3	15.0
TPU-Ac-BWH-40	283.9	314.1	415.9	354.5	22.1
TPU-Ac-BWH-60	258.7	300.4	408.9	383.4	28.6

**Table 4:** Selected mechanical properties of thermoplastic polyurethane and composites containing unmodified and modified buckwheat husks powder.

Material Code	Tensile Strength, MPa	Elongation at Break, %	Hardness, ShA
TPU-REF	8.5 ± 0.3	404.1 ± 6.7	26.3 ± 1.0
TPU-BWH-10	6.7 ± 0.4	107.2 ± 2.6	30.2 ± 1.0
TPU-BWH-20	8.1 ± 0.3	62.9 ± 3.2	36.7 ± 0.9
TPU-BWH-30	9.0 ± 0.1	65.1 ± 14.0	38.7 ± 1.7
TPU-BWH-40	10.5 ± 0.2	50.3 ± 8.3	45.4 ± 2.3
TPU-BWH-50	9.8 ± 1.3	19.3 ± 6.7	49.5 ± 1.6
TPU-BWH-60	10.9 ± 0.5	14.8 ± 0.5	54.6 ± 1.2
TPU-Ac-BWH-10	7.5 ± 0.1	288.4 ± 45.9	30.3 ± 1.2
TPU-Ac-BWH-20	6.4 ± 0.1	107.6 ± 22.2	34.8 ± 1.0
TPU-Ac-BWH-30	7.8 ± 0.3	50.9 ± 11.4	39.3 ± 1.1
TPU-Ac-BWH-40	8.4 ± 0.1	41.5 ± 3.0	44.1 ± 0.9
TPU-Ac-BWH-50	8.0 ± 0.3	15.9 ± 3.0	49.0 ± 0.7
TPU-Ac-BWH-60	10.4 ± 0.7	14.7 ± 0.9	56.8 ± 1.1

**Table 5:** Density, volume fraction of filler and polymer matrix-filler interaction parameters for obtained materials.

Material Code	Density, g cm^−3^	Volume Fraction of Filler (V_f_), %	Polymer Matrix–Filler Interaction Parameter (B)
TPU-REF	1.224 ± 0.001	-	-
TPU-BWH-10	1.234 ± 0.004	0.091	3.89 (R^2^ = 0.99)
TPU-BWH-20	1.245 ± 0.002	0.184	
TPU-BWH-30	1.262 ± 0.001	0.279	
TPU-BWH-40	1.278 ± 0.004	0.377	
TPU-BWH-50	1.289 ± 0.005	0.475	
TPU-BWH-60	1.301 ± 0.003	0.576	
TPU-Ac-BWH-10	1.235 ± 0.003	0.092	3.67 (R^2^ = 0.98)
TPU-Ac-BWH-20	1.246 ± 0.003	0.186	
TPU-Ac-BWH-30	1.258 ± 0.003	0.282	
TPU-Ac-BWH-40	1.269 ± 0.004	0.379	
TPU-Ac-BWH-50	1.280 ± 0.001	0.478	
TPU-Ac-BWH-60	1.294 ± 0.004	0.580	

## Data Availability

Data is contained within the article.
